# Role of Organo-Modifier and Metal Impurities of Commercial Nanoclays in the Photo- and Thermo-Oxidation of Polyamide 11 Nanocomposites

**DOI:** 10.3390/polym12051034

**Published:** 2020-05-02

**Authors:** Martina Ussia, Giusy Curcuruto, Daniela Zampino, Nadka Tzankova Dintcheva, Giovanni Filippone, Raniero Mendichi, Sabrina Carola Carroccio

**Affiliations:** 1 CNR-IMM, Via Santa Sofia 64, 95123 Catania, Italy; martina.ussia@ct.infn.it (M.U.); sabrinacarola.carroccio@cnr.it (S.C.C.); 2 CNR-IPCB, Via P. Gaifami 18, 95126 Catania, Italy; danielaclotilde.zampino@cnr.it; 3 Department of Civil, Environmental, Aerospace, Materials Engineering, University of Palermo, Viale delle Scienze, 90128 Palermo, Italy; nadka.dintcheva@unipa.it; 4 Department of Chemical, Materials and Production Engineering, University of Naples Federico II, Piazzale V. Tecchio 80, 80125 Naples, Italy; gfilippo@unina.it; 5 CNR- SCITEC, Via A. Corti 12, 20133 Milano, Italy; mendichi@ismac.cnr.it

**Keywords:** PA11, montmorillonite, Cloisite^®^ 30B, nanocomposites, photo-oxidation, thermo-oxidation, bio polyamide

## Abstract

The photo-oxidative degradation processes of bio-based PA11 nanocomposites containing montmorillonite (MMT) and the organo-modified Cloisite^®^30B were investigated to discriminate the influence of organo-modified components on the polymer durability. Indeed, despite the extensive studies reported, there are still ambiguous points to be clarified from the chemical point of view. To this aim, UV-aged materials were analyzed by Size Exclusion Chromatography (SEC), Inductively Coupled Plasma–Mass Spectrometry (ICP-MS) and Matrix-Assisted Laser Desorption Ionization Time-of-Flight Mass Spectrometry (MALDI-TOF MS). This enabled determining changes in both chemical structure and Molar Masses (MMs) induced by light, heat and oxygen exposure. The addition of organo-modified nanoclays strongly affected the PA11 light durability, triggering the macromolecular chains scission due to the typical αH, Norrish I and II mechanisms. However, the main contribution in boosting the photo-oxidative degradation is induced by iron impurities contained into the clays. Conversely, thermo-oxidation process performed at 215 °C was unambiguously affected by the presence of the organo-modifiers, whose presence determined an enhancement of crosslinking reactions.

## 1. Introduction

Polyamide-11 (PA11) obtained from castor beans is a promising material as a “green” alternative in a variety of applications ranging from household devices to engineering materials [1]. By now, PA11 finds excellent placings in metal coatings, flexible pipes for automotive, and offshore applications, and the global castor oil and derivates market is expected to reach USD 1.81 billion by 2020. However, it is well known that polyamides (PAs) are not intrinsically stable in the presence of oxygen and/or humidity, especially at high temperature. In particular, PA11 undergoes degradation during processing and in-service applications, experiencing a drastic drop in terms of mechanical and physical properties [2,3,4]. To mitigate these drawbacks and possibly spread out the use of PA11 as a viable green alternative to traditional polymeric materials, one opportunity is the addition of nanofillers such as clays, graphene derivates and metal oxides, which provide specific properties depending on the needed related to the field of application [5,6,7]. Specifically, nanoclays determine several benefits, such as a significant increase in thermal stability, gas barrier, mechanical properties [8,9] as well as flame retardancy [10]. Furthermore, a growing interest of polymer based nanoclays was recently manifested in the area of environmental and medical applications [11], where the use of biobased PA11 could make the difference. To obtain all these achievements, a uniform and fine distribution of nanofillers into the polymer matrices is required. The extremely high polymer-nanoparticle interface sought in polymer nanocomposites can imply the insurgence of severe degradation phenomena. The durability of PA11 manufactured articles is compulsory especially for outdoor applications, and, regarding this point, the addition of nanoclays may strongly modify their photooxidative resistance [12].

Controversial results regarding the UV light resistance of polymer based nanoclays were reported in the literature, and the mechanisms that trigger the acceleration of the oxidative processes are also still debated. Several papers reported an adverse influence of organo-modified MMT nanofillers, concluding that nanocomposites degrade faster than the neat polymers [12,13]. The origin of degradation is mainly associated to the presence into the clays of metal ion impurities, which catalyze the hydroperoxides decomposition by accelerating the entire photo oxidative process [12,13,14,15]. Other works ascribed this phenomenon to the presence of alkylammonium modifiers [16,17,18,19], whose decomposition can release α olefins, tertiary amines and 1 chloroalkanes, able to catalyze the degradation process [20,21,22]. In particular, that presence of free amines was proposed to play a significant role in the scission of peptide linkage. In addition, Larchè et al. suggested as a source of photo-oxidation of some acrylic-urethane thermoset networks the presence of chromophores impurities, which absorb UV light producing radicals [23]. Given that, and considering the increasing interest on PA11 nanocomposite materials, the goal of this paper is to gain insight into the degradation pathways in PA11-nanoclay nanocomposites, shedding light on the role of each component of the filler to the photodegradation process. We use an original “molecular” approach to determine and differentiate the contribution of organo-modifiers and iron impurities on degradation of PA11 matrix. To our knowledge, this strategy was not reported before to establish the role of nanoclays in photo-degradation processes. Indeed, the possibility to observe in depth structural modifications of polymeric chains correlating them to specific factors, can give the unprecedented opportunity to better recognize the behavior of degradation mechanisms involved. Using such a multidisciplinary approach enabled us to highlight limits or benefits in adding nanoclays to PA11 for outdoor applications. Specifically, the contributions of iron impurities and organoclay modifier to the photo-oxidation of PA11 has been explored. In addition, thermo-oxidative degradation was also investigated for enriching the conclusions of a previous study [24]. Most of the degradation studies regarding polymer nanocomposites were focused to evaluate change in mechanical properties, whereas here the focus is on the chemical aspect. To this purpose, MALDI MS was used to investigate changes in macromolecular structures. Specifically, the formation of peculiar degradation products even in trace amounts were detected and correlated to SEC data. The results obtained for the systems so far investigated by MALDI are remarkably highly informative [25,26]. Indeed, precise information on the size, structure and end groups of molecules originated from the oxidation process can be collected. 

## 2. Background

Degradation mechanisms occurring in PAs during thermal, thermo- and photo-oxidative processes are well established in the literature [2,4,27,28,29,30,31]. Nevertheless, targeted studies on degradation mechanisms of PA11 and the related change in macromolecular structures are very scarce if compared with the most renowned PA6 and PA66 materials. Furthermore, the structural identification of products present in the PA11 matrix is based on the detection of volatile low molecular weight compounds by Py/GC-MS or GC-MS techniques, as well as by averaging spectroscopic methods (^1^H-NMR, UV) [3,27,28].

**Figure 1:**
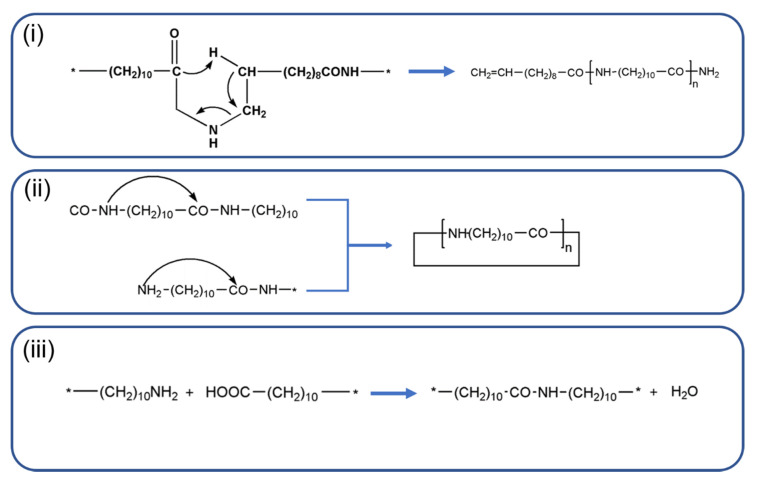
Main thermal processes occurring in PA11 during degradation.

The main thermal processes occurring in PA11 during degradation at temperature higher than 180 °C involve at least: (i) the β CH hydrogen transfer with the formation of oligomers having amide (CONH_2_) and olefinic terminal groups; (ii) exchange reactions with the production of cyclic species; (iii) post condensation reactions trough OH and NH_2_ terminal groups (Figure 1). 

**Figure 2:**
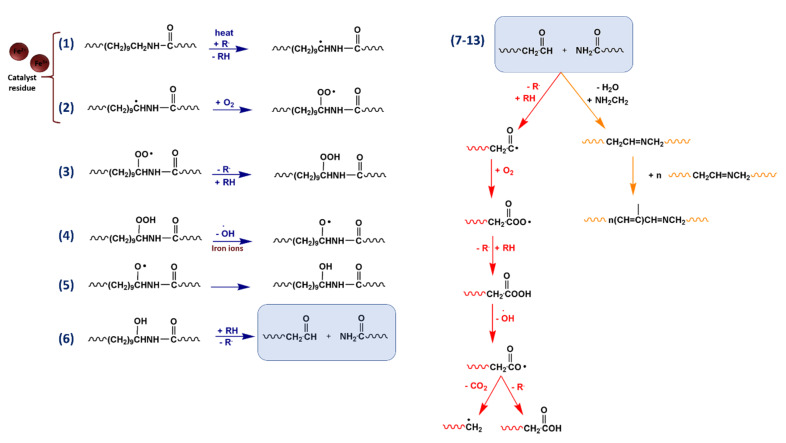
The oxidative pathway via the α-CH hydrogen abstraction.

At high temperature, the presence of air determines the concomitantly activation of other oxidative routes. The oxidative pathway via α-CH hydrogen abstraction involving the reactive α amino methylene is reported in Figure 2. In this case, degradation leads to the formation of stable amide (CONH_2_) and aldehydes terminal groups, which can be further oxidised to carboxylic acid.

**Figure 3:**
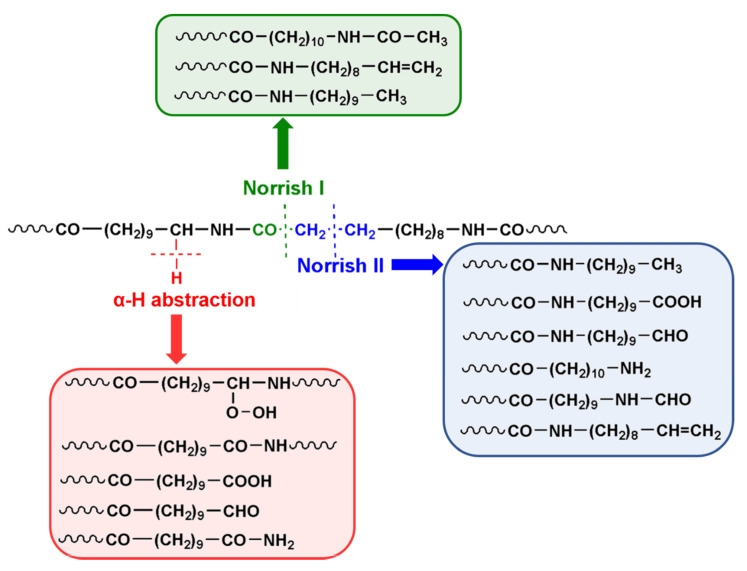
Photo-oxidation processes in PA11.

The amide terminal groups produced during both thermal and thermo-oxidative processes could subsequently undergo to dehydratation forming nitrilic species. In addition, taking into account the mechanism postulated by Karstens and Rossback for PA6 [32], azomethyne groups could be formed. The latter species can produce unsaturated oligoenimine structures responsible for the color formation and insoluble residue material (Figure 2). PA11 photo-oxidation pathways are reported in the Figure 3. In common with the aforementioned thermo-oxidative pathway (Figure 2, reactions 1–6), photo-oxidation involves the formation of stable amide (CONH_2_) end groups derived from light induced α-H abstraction mechanism. As evidenced also for other polymer matrices included PAs, degradation starts out with α-H abstraction mechanism as detected by MALDI analysis [30,31]. Since PAs adsorb up to 250 nm, photooxidative activation (reactions 1 and 2 in Figure 2) occurring at higher wavelengths, is promoted by the presence of chromophores impurities into the polymer matrix [33]. Among them, metal ions deriving from catalyst and/or nanoclays addition, can play a key role in absorbing light, triggering the initial formation of organic radicals. Furthermore, metallic species can also promote the decomposition of polymer hydroperoxides to form reactive radicals [17]. Thus, if compared with unfilled samples, nanocomposites based on nanoclays containing iron ions experience accelerated aging and, hence, faster depletion of mechanical properties. Indeed, light irradiation of iron species can promote the transition of their electrons from the valence band to the conduction band. Here, excited electrons can be transferred to chemisorbed oxygen on the nanoclays surface generating superoxide radicals. At this point, the latter can react with polymers preferring the weaker bonds of macromolecular chains, specifically the α-CH hydrogen linkage. Of course, other photocatalytic processes can be involved and their occurrence is not excluded [34].

PA11 photo-oxidation pathways are reported in the Figure 3. The formation of stable amide (CONH_2_) end groups derived herein from α-H abstraction mechanism is highlighted. As evidenced also for other polymer matrices included PAs, degradation starts out with α-H abstraction mechanism as detected by MALDI analysis [30,31]. At prolonged exposure times, NORRISH I and NORRISH II photo cleavages turn out to be active, further promoting chain scission with the formation of peculiar end groups detectable from MS analysis [30,31,35].

## 3. Materials and Methods

### 3.1. Materials

2-(4hydroxyphenylazo) benzoic acid 0.1 M, hexafluoroisopropanol (HFIP), PA11, unmodified montmorillonite (MMT) and organo-modified MMT known as Cloisite^®^30B were commercial products. Specifically, natural MMT possesses a particle size in the range of 30–90 µm. The organo-modifier of Cloisite^®^30B is bis-(2-hydroxyethyl) methyl tallow alkyl ammonium cations, and the organic mass content is about 28%. More specific Cloisite^®^30B characteristics are given in Table 1.

### 3.2. PA11 Nanocomposites Preparation

Nanocomposites at 3 and 9 weight % of filler were prepared by melt compounding using a DSM Xplore micro-compounder. Extrusion leaded to a strand which was subsequently granulated to obtain pellets with diameter of about 3 mm and comparable length. Specifically, the pellets and the filler in powder were dried over night at 90 °C under vacuum and loaded simultaneously into the mixing apparatus. All nanocomposites were extruded under gaseous nitrogen flow at T = 215 °C and 80 rpm. The sample are coded “PA11-XCY”, where *X* identifies MMT or Cloisite^®^30B (C), and *Y* is the percentage of filler. Pure PA11 used as reference material was extruded under the same conditions.

### 3.3. Photo-Oxidative Degradation of PA11 Samples

Photo-oxidation was carried out by irradiating (0.68 W/m^2^) the extruded composites on a QUV PANEL apparatus at 60 °C by exposing them to UVA 340 lamps up to 5 days. Virgin PA11 was used as a standard. Before the exposure, all pellets were cut obtaining samples with similar length and weight of ~10 mg. Then, each sample was dissolved in the appropriate solvent for further analyses.

### 3.4. Molecular Characterization by SEC-MALS Chromatographic System

The molar mass distribution (MMD) of polymers was determined by a multi-detector size exclusion chromatography (SEC) using two on-line detectors: (i) a multi-angle light scattering (MALS); (ii) a differential refractometer (DRI). The SEC-MALS system consists of an Alliance 2695 separation module from Waters with a serial setup Alliance-MALS-DRI.

### 3.5. Thermogravimetric Analysis

TGA measurements on PA11 and PA11 nanocomposites were performed using a thermogravimetric apparatus (TA Instruments Q500) under 60 mL min^−1^ of synthetic air flow with a heating rate of 10 °C min^−1^ from 40 to 800 °C (see Supporting information).

### 3.6. Wide Angle X-Ray Diffractometry (WAXD)

WAXD were performed using a Siemens D-500 diffractometer with Cu Kα radiation (wavelength 0.154 nm). The 2θ interval 2–10° was investigated with step size of 0.02°.

### 3.7. Transmission Electron Microscopy (TEM)

TEM were performed by using a Philips CM 200 apparatus. The observed samples were thin slices (thickness ∼100 nm) cut at room temperature from the as-extruded pellets.

### 3.8. MALDI-TOF MS Analysis

MALDI-TOF mass spectra were recorded in positive ion mode through Voyager-DE STR (*Applied Biosystems*) mass spectrometer furnished of nitrogen laser (337 nm) with a 3-ns pulse width and accelerating voltage of 20 kV. 2-(4 hydroxyphenylazo)benzoic acid 0.1 M in hexafluoroisopropanol (HFIP) was used as matrix, mixed with different volumes of polymer solution (5 mg/mL in HFIP).

### 3.9. Inductively Coupled Plasma–Mass Spectrometry (ICP-MS)

Quantitative determination of metal ions in solution after sequestration procedure was performed by ICP-MS analysis using a NEXION 300X (PerkinElmer). Then, 10 ppb of internal standard (multielement standard solution for ICP-TraceCERT^®^) was added. Table 2 reports data obtained for the sample PA11, PA11-MMTC3 and -MMTC9, PA11-CC3 and -CC9.

## 4. Results and Discussion

### 4.1. Molar Masses Determination

**Figure 4:**
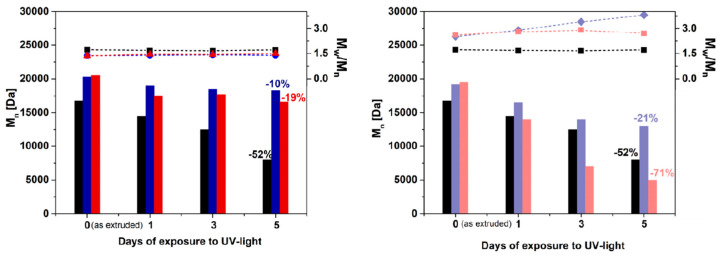
Evolution of molecular masses (bars, left axis) and polydispersity index (dots, right axis; dashed lines are a guide for the eye) as a function of UV-light exposure time for PA11 (black), PA11-CC3 (blue), PA11-CC9 (red), PA11-MMTC3 (violet) and PA11-MMTC9 (rose).

The evolution of average number molecular weights (M_n_) and polydispersity index (PDI = M_w_/M_n_) as a function of the exposure time is shown in Figure 4a,b for pure PA11 and the prepared nanocomposites. The obtained SEC data were averaged from the presence of non-degraded bulk material, being only the surface exposed to the UV-light. Before UV-light exposure (day zero in Figure 4a,b), an initial increment of M_n_ respect to pure PA11 is registered for the both sets of composites based on Cloisite^®^ 30B and MMT. This is likely due to post-polymerization reactions that occur during melt compounding at 215 °C (see Figure 4). The M_n_ of pure PA11 progressively decreases during UV exposure, roughly halving in 5 days. No appreciable effects were instead found on the PDI, which remains stable around 1.6.

Looking at the effect of the nanoparticles, a decrement of M_n_ was noticed for all the samples without the production of insoluble gel residue, but the extent of degradation depends on the nature of the filler. In particular, the comparison of the M_n_ values shown in Figure 4a,b reveals a percentage decrease of Mn much smaller than that of pure PA11, with decreases of 10% and 19% for PA11-CC3 and PA11-CC9, respectively, after 5 days of UV-light exposure. These data indicate a stabilizing action of the Cloisite^®^30B nanoclays. Specifically, the beneficial effect of the nanoparticles is ascribed to the physical shielding of the clay lamellae, which preserve the fraction of intercalated polymer from UV-light exposure [24]. It is important to observe that concurrent nanoparticle-related chemical degradation pathways cannot be excluded and could compete with the shielding effect of the clay lamellae. This is evident in the samples based on unmodified MMT, in which a very limited polymer chains intercalation is expected. As a result, a depletion of UV light resistance was noticed, especially at high MMT amount. Indeed, as shown in Figure 4b, the percentage reduction of M_n_ in the sample PA11-MMTC9 after 5 days of irradiation is higher than in pure PA11. Further evidence of MMT-induced degradation phenomena emerges from the analysis of the PDI, which increases suggesting the occurrence of random chain scission phenomena even in the sample PA11-MMTC3, which yet exhibits higher M_n_ than pure matrix.

**Figure 5:**
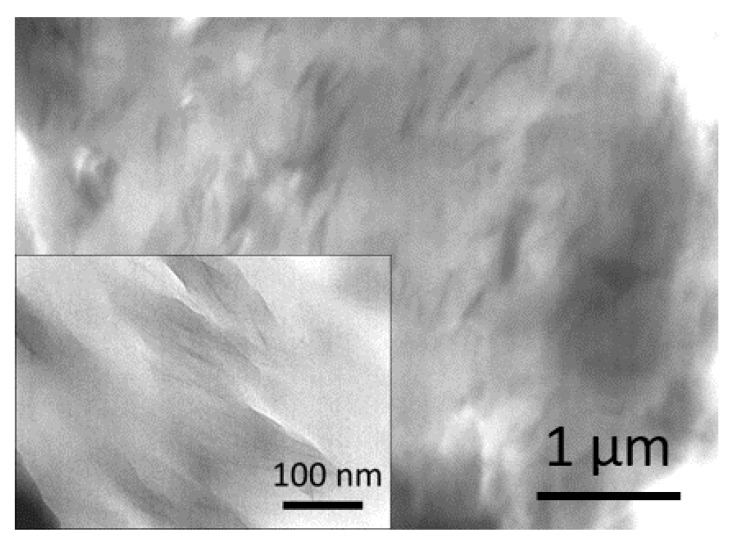
TEM micrograph of the sample PA11-CC9; the inset shows a magnification of exfoliated clay lamellae.

The trend observed for Cloistite^®^ 30B is in agreement with data reported in literature [24] and indicates that the presence of organo-modifier preserves the fraction of intercalated polymer from UV-light exposure. Such a mechanism is expected to be play a major role in case of exfoliated samples, in which large fractions of polymer are surrounded by clay lamellae. The WAXD spectra of Cloisite^®^ 30B and PA11-CC3 and CC9 reported in Figure S1 shows the disappearance of the d001 diffraction peak of pure nanoclay, meaning that the ordered structure of the layered filler is lost in the nanocomposites. The good degree of dispersion of the nanoclay is also evident in Figure 5**,** where representative TEM micrographs of the sample PA11-CC9 are reported.

**Figure 6:**
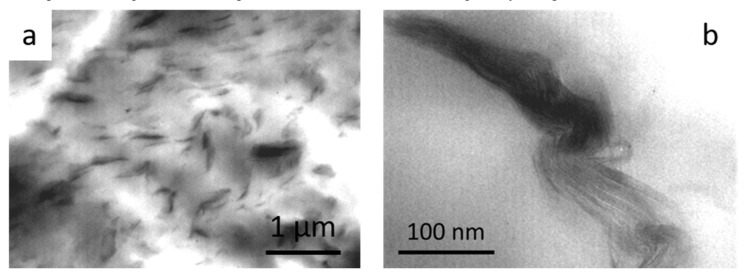
(**a**) TEM micrographs of the sample PA11-MMTC9; a magnification of an unmodified clay stack is shown in (**b**).

It is important to observe that concurrent nanoparticle-related chemical degradation pathways cannot be excluded and could compete with the shielding effect of the clay lamellae. This is evident in the samples based on unmodified MMT, in which a very limited polymer chains intercalation was (see Figure 6). A depletion of UV light resistance was thus noticed, especially at high MMT amount.

On the other hand, it should be kept in mind that the presence of the alkylammonium organomodifier into the polymer matrix is known to promote degradation by Hoffman elimination at temperature above 180–200 °C [36]. Obviously, this kind of reaction does not take place during the photo-process carried out at 60 °C. At the same time, the significant photo-degradation phenomena noticed in the samples containing pure MMT prove that other degradation pathways can occur irrespective of organo-modifier, as will be discussed in the next sections.

### 4.2. MALDI-TOF Analyses

To identify the degradation products of PA11 in the presence of MMT or Cloisite^®^ 30B, MALDI-TOF MS analysis was performed on samples of photo-oxidized materials, collecting and analysing them at different aging times. The MALDI spectrum of PA11 was already reported in literature [12,13], and an expanded portion (from 1304 to 1346 *m/z*) is reported in Figure S2 together with the relative assignments. As expected, the presence of cyclic and linear chains terminated with NH_2_/COOH end-groups were noticed [9,30,31]. After exposure to UV-light, peaks related to the occurrence of the αH-abstraction, Norrish 1 and 2 photo-oxidation mechanisms were also detected [9,30,31]. Nevertheless, their appearance was found to depend on the exposure time and nanoclays content, as well [4,9,24]. As consolidated in previous works [30,31] the appearance in the MALDI spectra of CONH_2_/NH_2_ terminal groups, indicates the initiation of degradation process. Specifically, as aforementioned, this degradation products can be derived from thermo, thermo-oxidative and photo-oxidative pathways depending on the source used to degrade the material.

**Figure 7:**
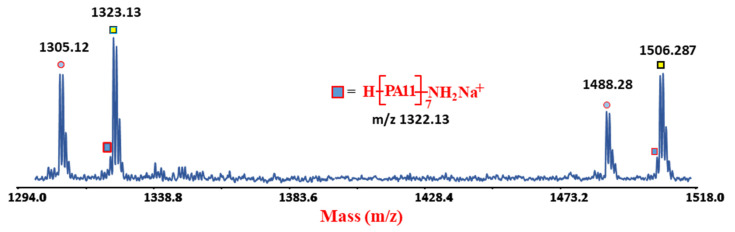
Expanded portion of MALDI spectrum obtained in reflectron and positive mode of extruded PA11-MMTC3 sample.

The MALDI spectra of both sets of the as-extruded nanocomposites revealed the appearance of peaks having a very low intensity, assigned to CONH_2_/NH_2_ terminal groups (Figure 7).

The formation of this peak related to a specific degradation product (Figure 2) bearing CONH_2_/NH_2_ terminal groups confirms the occurrence of thermo-oxidative reactions at the operation condition used to extrude the materials. These species, not visible in the MALDI spectrum of neat PA 11 (see SI), indicates that the addition of nanoclays triggers part of chains scission during the processing, although SEC values registered for the same samples revealed a clear increment of the molecular parameters. This apparently controversial data can be rationalized by considering that PA11 post-polymerization is the main reaction during extrusion able to prevail the limited chain scission process occurred in the melt state. The latter phenomenon can be revealed from the high sensitivity of MS non-averaged technique, whereas SEC failed providing only the final averaged increment of MMs values [25,26].

**Figure 8:**
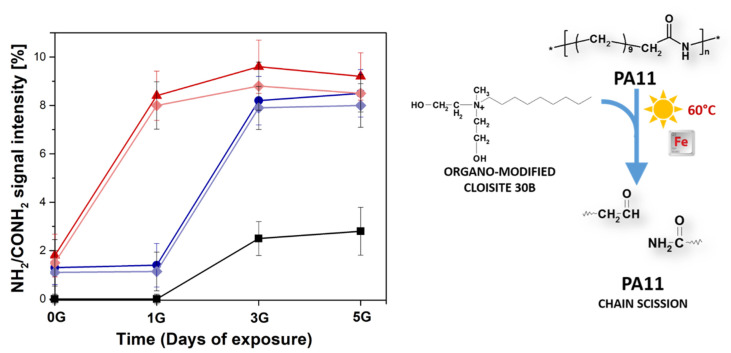
Intensities of Matrix-Assisted Laser Desorption Ionization (MALDI) peak related to NH_2_/CONH_2_ oligomers plotted as a function of exposure time for PA11 (black square), PA11-CC3 (blue circle), PA11-CC9 (red up triangle), PA11-MMTC3 (violet diamond) and PA11-MMTC9 (rose diamond) samples.

Once all materials (PA11 and PA11 nanocomposites) are exposed to UV light, the formation of these products are selectively accomplished by the photooxidative process as described in Figure 3. The intensities of diagnostic peak at m/z 1322.33 corresponding to CONH_2_/NH_2_ oligomers are plotted in Figure 8 as a function of the exposure time as obtained from MALDI spectra.

The unfilled sample showed the increase of this diagnostic peak only after three days of exposure. Differently, both PA11-CC3 and PA11-CC9 revealed the same degradation peaks deriving from chains cleavage after only 1 day, showing higher intensities values compared to neat PA11. Remarkably, this scission mechanism is the same involved in the first stage of PA11 thermo-oxidation pathway a 215 °C, but in this case cross-linking phenomena triggered by organo-modified nanoparticles took place [24]. As indicated in the literature [36], the acceleration of such reaction is due to Hofmann elimination of quaternary ammonium salts occurring at temperature above 180 °C [35]. The produced α-olefins and aldehydes participate to the acceleration of the thermal-oxidation process, boosting up the insoluble gel formation [4,24]. Conversely, photo-exposure process performed at 60 °C was not able neither to induce gel formation or to trigger Hofmann elimination. As far as this point is concerned, it is reasonable to suppose that the acceleration of the photo-oxidation process is driven by the iron impurities into nanoclay structures [2,14,35], whose presence was confirmed by ICP-MS analyses (see Table 2). Further proof of the key role of iron impurities comes from the analysis of the MMT-based samples, which exhibit the same trend as the Cloisite^®^ 30B-filled samples sharing comparable iron content.

Now, let us recall the thermo-oxidation process of PA11 nanocomposites, comparing it with the present results obtained from the photo-aging. Clearly, the contribution of metal impurities to the auto-oxidation cannot be excluded. However, the combination of high temperatures, air and α-olefins originated by alkyl ammonium modifier promoted cross-linking reactions via peroxides formation. It is important to highlight that the pure thermal processes of PA11-CC3 and PA11-CC9 at 215 °C did not produce gel formation up to 150 min [4]. This means that oxygen is a determining factor in generating the insoluble residue, whose formation can be accelerated from the presence of iron or organo-modifiers.

To discriminate the role of each aforementioned component in promoting crosslinking reactions, thermo-oxidation of PA11, PA11-MMTC9 and PA11-CC9 were performed at 215 °C. The as-degraded materials were solubilized in HFIP analyzing their soluble part by MALDI, whereas the insoluble part was washed several times with HFIP, dried and weighted.

**Figure 9:**
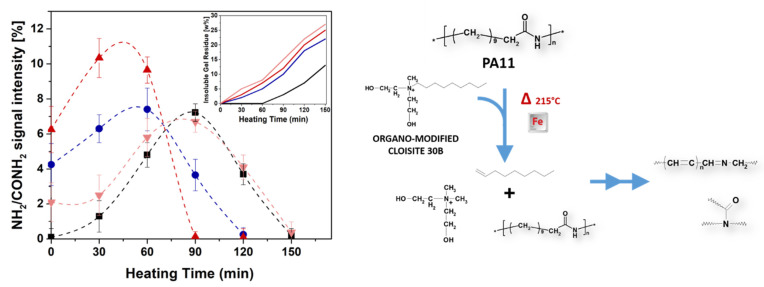
Intensities MALDI peak related to NH_2_/CONH_2_ oligomers for PA11 (black square), PA11-CC3 (blue circle), PA11-CC9 (red up triangle) and PA11-MMTC9 (rose down triangle) samples, plotted as a function of the heating time. The weight percentage of insoluble gel residue produced after 150 min of heating time for each composite are reported in the inset.

The time-evolution of NH_2_/CONH_2_ species, strictly connected to the peroxide formation and thus crosslinking reactions (Figure 2), is reported in Figure 9 together with the residue amounts formed at 215 °C. Even though a residue was present in the polymer filled with MMT, the relative percentage lower than that found in PA11-CC9 sample for all collected heating time. The amount of insoluble residue measured for both PA11-CC3 and PA11-CC9 nanocomposites clearly suggests an active contribution of bis(2-hydroxylethyl) methyl tallow alkyl ammonium compounds of Cloisite^®^ 30B organo-modifier in boosting crosslinking reactions. In agreement with our previous assumption, α-olefins produced from their Hofmann elimination at 215 °C are very susceptible to air and moisture, producing oxygenated species as final products. It was suggested that the formation of α-olefin hydroperoxides is accomplished by the capture of available hydrogens during initial step of auto-oxidation. The favorable point of attack is clearly the α-amino methylene position of PA11 chains. At this point, the well-known α-CH hydrogen abstraction mechanism proceeds, thus accelerating the formation of insoluble residue.

## 5. Concluding Remarks

The influence of MMT and organo-modified Cloisite^®^30B on the photo and thermal oxidation processes of PA11 nanocomposites was investigated by using SEC and MALDI MS analyses. The combination of different “molecular” analytical techniques and the comparative analysis of unmodified and organo-modified nanoclays enabled to discern among the different contributions of each component of the filler to the degradation processes in PA11 nanocomposites. Data collected suggested a contrasting role of organo-modified clays, which on the one hand acts as a physical shielding that preserves the intercalated polymer from photo-oxidation, and on the other hand plays an active role in promoting degradation. An active and predominant contribution of iron impurity into the nanoclays in determining the chains scission during photo-aging at 60 °C clearly emerged. At the same time, MALDI measurements collected for thermo-oxidised PA11 nanocomposites highlighted an active involvement of organo-modifier in degrading polymer, increasing also the formation of insoluble gel during the process.

## Figures and Tables

**Table 1:** General characteristics of Cloisite^®^ 30B and unmodified Cloisite^®^ Na+ by Southern Clay Products.

Organoclay	Modifier Concentration	Specific Gravity (g/cc)	Particle Size (µm)	Interlayer Spacing (*d*_001_)
Cloisite^®^30B	90 mEq/100 g clay	1.98	13	1.85
Cloisite Na+	-	2.86	≤2	1.17

**Table 2:** Iron content (ppb) calculated by ICP-MS for PA11 nanocomposites.

	PA11-MMTC3	PA11-MMTC9	PA11-CC3	PA11-CC9
**Iron Content** **(ppb)**	105.0	183	104.5	181.8

## Supplementary Materials

The following are available online at https://www.mdpi.com/2073-4360/12/5/1034/s1, Table S1: TGA measurements on PA11, PA11-CC3, PA11-CC9, PA11-MMTC3 and PA11-MMTC9 in presence of air, Figure S1: WAXD spectra of Cloisite® 30B and nanocomposite samples PA11-CC3 and CC9, Figure S2: Expanded view of the 1294–1346 m/z region of MALDI-TOF Mass spectrum of PA11 recorded in positive reflectron mode by using HABA matrix.
