# Supplementary material for: Role of Organo-Modifier and Metal Impurities of Commercial Nanoclays in the Photo- and Thermo-Oxidation of Polyamide 11 Nanocomposites
Source: Polymers (Basel). 2020 May 2;12(5):1034. doi: 10.3390/polym12051034 (PMC7284445; doi:10.3390/polym12051034)
Supplement: Supplementary file 1 [file polymers-12-01034-s001.pdf]

Role of organo-modifier and metal impurities of commercial nanoclays in the photo- and thermo-oxidation of Polyamide 11 nanocomposites

Martina Ussia[1], Giusy Curcuruto[2*], Daniela Zampino[2], Nadka Tzankova Dintcheva[3], Giovanni Filippone[4], Raniero Mendichi[5] and Sabrina Carola Carroccio[1,2]

[1] CNR-IMM, Via Santa Sofia 64, 95123 Catania, Italy

[2] CNR-IPCB, Via P. Gaifami 18, 95126 Catania, Italy

[3] University of Palermo, DICAM, Viale delle Scienze, 90128 Palermo, Italy

[4] University of Naples Federico II, Piazzale V. Tecchio 80, 80125 Naples, Italy

[5] CNR- SCITEC, Via A. Corti 12, 20133, Milano, Italy

Thermogravimetric analysis

TGA measurements on PA11, PA11-CC3 and PA11-CC9 were performed in presence of air using a thermogravimetric apparatus (TA Instruments Q500) under air flow (60 mL min^{-1}) with a heating rate of 10 °C min^{-1} from 40 to 800 °C. The obtained results were summarized in **Table S1**. As expected, the collected temperatures at the maximum derivative of weight loss (T_d) increased, displaying values of 395.2, 408.6 and 409.1°C for PA11, PA11-CC3 and PA11-CC9, respectively.

Table S1.

TGA measurements on PA11, PA11-CC3, PA11-CC9, PA11-MMTC3 and PA11-MMTC9 in presence of air.

Sample	$T_{\Delta m=5\%}$ (°C)^a	$T_{\Delta 1=5\%}$ (°C)^b	% R^c
**PA11 extruded (T0)**	395.2	441.7	1.33
**PA11-CC3 (T0)**	408.6	462.6	3.21
**PA11-CC9 (T0)**	409.1	463.8	4.20
**PA11-MMTC3**	398.5	440.6	3.00
**PA11-MMTC9**	399.2	440.9	3.98

[a] *Onset temperature for decomposition (5% loss of initial weight)*

[b] *Decomposition maximum temperature*

[c] *Weight residue*

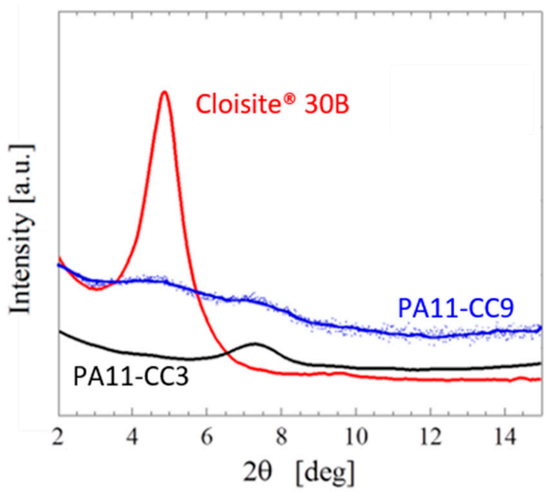

Figure S1. WAXD spectra of Cloisite® 30B and nanocomposite samples PA11-CC3 and CC9.

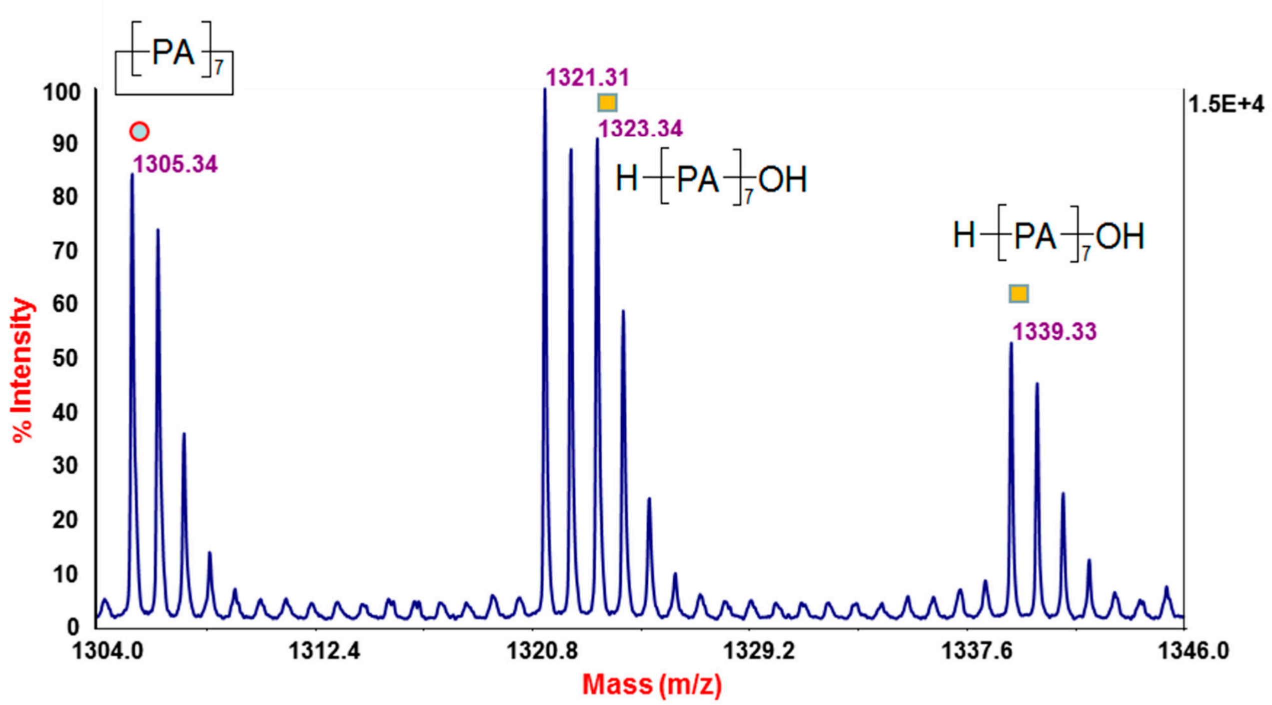

Figure S2. Expanded view of the 1294-1346 *m/z* region of MALDI-TOF Mass spectrum of PA11 recorded in positive reflectron mode by using HABA matrix.
